# Effects of Light Spectral Quality on Photosynthetic Activity, Biomass Production, and Carbon Isotope Fractionation in Lettuce, *Lactuca sativa* L., Plants

**DOI:** 10.3390/plants11030441

**Published:** 2022-02-05

**Authors:** Ivan G. Tarakanov, Daria A. Tovstyko, Maxim P. Lomakin, Alexander S. Shmakov, Nikolay N. Sleptsov, Alexander N. Shmarev, Vladimir A. Litvinskiy, Alexander A. Ivlev

**Affiliations:** 1 Department of Plant Physiology, Russian State Agrarian University—Moscow Timiryazev Agricultural Academy, Timiryazevskaya Str., 49, 127550 Moscow, Russia; tov.dasha@mail.ru (D.A.T.); max124c41@gmail.com (M.P.L.); 5456685@gmail.com (A.S.S.); inkss@mail.ru (N.N.S.); aa.ivlev@list.ru (A.A.I.); 2 Institute of Basic Biological Problems, Russian Academy of Sciences, 142290 Pushchino, Moscow Region, Russia; shurik_bx_04@mail.ru; 3 Borissiak Paleontological Institute, Russian Academy of Sciences, 123, Profsoyuznaya Str., 117647 Moscow, Russia; vl.litvinskiy@gmail.com

**Keywords:** *Lactuca sativa*, LEDs, plant factory, photosynthesis, chlorophyll fluorescence, carbon isotope discrimination

## Abstract

The optimization of plant-specific LED lighting protocols for indoor plant growing systems needs both basic and applied research. Experiments with lettuce, *Lactuca sativa* L., plants using artificial lighting based on narrow-band LEDs were carried out in a controlled environment. We investigated plant responses to the exclusion of certain spectral ranges of light in the region of photosynthetically active radiation (PAR); in comparison, the responses to quasimonochromatic radiation in the red and blue regions were studied separately. The data on plant phenotyping, photosynthetic activity determination, and PAM fluorometry, indicating plant functional activity and stress responses to anomalous light environments, are presented. The study on carbon isotopic composition of photoassimilates in the diel cycle made it possible to characterize the balance of carboxylation and photorespiration processes in the leaves, using a previously developed oscillatory model of photosynthesis. Thus, the share of plant photorespiration (related to plant biomass enrichment with ^13^C) increased in response to red-light action, while blue light accelerated carboxylation (related to ^12^C enrichment). Blue light also reduced water use efficiency. These data are supported by the observations from the light environments missing distinct PAR spectrum regions. The fact that light of different wavelengths affects the isotopic composition of total carbon allowed us to elucidate the nature of its action on the organization of plant metabolism.

## 1. Introduction

The application of light-emitting diodes (LED) in horticultural lighting systems provides new possibilities for light intensity and light spectrum fine regulation along with a significant reduction in energy consumption [1,2,3]. A breathtaking possibility to modulate the LED lighting spectrum can also help in promoting the accumulation of important plant metabolites, which are often associated with nutraceutical properties, as has been shown in various crops, including lettuce [4]. The set-up of plant-specific light protocols for their cultivation is a critical phase in improving the sustainability of indoor growing systems [2].

Besides photosynthesis, plants are capable of perceiving and processing information with light signals from their biotic and abiotic surroundings for optimal growth and development [5]. Reviews of studies on light quality effects on plant growth and development can be found elsewhere [6,7,8]. Red and blue are generally recognized as the most important light regions necessary for plant development and growth [3]. However, other wavelengths (such as those corresponding to yellow or green colors) could also have a role in affecting the quality of crops [6]. Blue light is involved in a wide range of plant processes such as phototropism, photomorphogenesis, stomatal opening, and leaf photosynthetic functioning [9]. Most studies assessing the effects of blue light (blue LEDs) on the leaf or whole plants have either compared their response to a broadband light source with the response to blue-deficient light [10] or plants grown under red light alone [11,12]. On the other hand, red LEDs emit a narrow spectrum of light (660 nm) that is close to the maximum absorbance for both chlorophyll and phytochromes. The absorption of blue and red light (LEDs) by plants has been measured as 90% [13], which indicates that plant development and physiology is strongly influenced by blue and red light [6]. The effects of green light tend to reverse the processes established by red and/or blue light. In this way, green light may be functioning in a manner similar to far-red light, informing the plant of photosynthetically unfavorable conditions and triggering adaptative responses [14]. Many studies have been reported on several crops grown under deficiency/efficiency or using a combination of red and blue light at different wavelengths [15,16] to investigate their effects on plant growth and development. While red light promotes biomass accumulation, growth, and photosynthesis in lettuce, blue LED light is effective in stimulating photomorphogenesis and adaptive phenomena such as the stomata-opening/closing-regulation mechanism, as well as biomass accumulation and chlorophyll and anthocyanin biosynthesis [3,17]. A positive growth response to the combination of blue and red light was confirmed in Batavia lettuce plants [18]. Green LED light regulates leaf expansion, stem stretching, and stomatal conductance. Moreover, it has been shown that green LED light addition leads to greater dry mass accumulation and growth stimulation [19].

The plant perceives light environment signals by means of photosynthetic apparatus (PSA) and specific photoreceptors sensitive to different light spectral regions. Blue and red light are not equal in their effects on photoreceptors: red light is perceived in addition to PSA by phytochromes only, and blue light is absorbed by both phytochromes and blue-light receptors (cryptochromes, phototropins) [20]. Blue light influences a greater number of photoreceptors and is functionally more versatile. It is most effective in stimulating the transcription of photosynthesis-related genes (via cryptochromes and phytochromes) [21]. Interestingly, barley plants grown with monochromatic red light demonstrated specific organization of chloroplast membranes (shaggy-formed grana) and light-harvesting complexes (increased energy transfer to PSI, possibly due to spillover promoted by this particular granum structure) [20]. These specific responses can be related to contradictory information from the photoreceptors; the signals from the phytochromes and photosynthetic apparatus indicate the incidence of light, while the lack of a signal from the blue-light receptors can be misinterpreted as darkness [20]. Most of the negative monochromatic red-light effects can be avoided by the addition of blue light [22,23,24]. Furthermore, a combination of red and blue light in certain cases can result in synergetic effects in biomass accumulation [25,26] Plant photosynthesis and growth, directly or indirectly, can also be mediated by the photoreceptor response. Additionally, chloroplasts play an important role in photoreceptor-mediated control of photomorphogenic responses [27]. The main obstacle in the transition to LED lighting in crop production is that it involves a complex system change beyond lighting (e.g., plant light recipes, which are species- and often cultivar-dependent), resulting in serious associated costs [28]. Lighting systems using specific wavelengths are capable of target compound biosynthesis fortification; however, special attention has to be paid to the stress the artificial light may cause in the photosynthesis and biomass accumulation [29]. To explore the action mode of different light spectrum regions, various experimental approaches are used. Thus, in the studies on the blue-light effects, plant responses to a broadband light source with a response to blue-region-deficient light were compared [10] with plants grown under red light alone [11]. So, the experimental set up can include studies on the effects of monochromatic irradiation. Additionally, plant responses to photosynthetically active radiation (PAR) missing distinct spectrum regions can be investigated [30].

In our studies with lettuce plants, we have used both screens mentioned above, emphasizing research on light spectral quality effects on the carbon isotope composition of plant biomass (Section 2.3). It is known that plant cells are able to fractionate carbon isotopes in the light and in the dark [31,32]. The carbon isotope composition of plant leaf biomass is mainly related to the light processes, CO_2_ assimilation, and photorespiration [32,33]. The ^12^C enrichment of plant biomass during CO_2_ assimilation occurs at Calvin cycle entry during RuBP carboxylation. During photorespiration, carbon isotope fractionation occurs with the opposite sign, thus reducing the effect of CO_2_ assimilation and enriching biomass with ^13^C. The isotope effect of photosynthetic assimilation and photorespiration are coupled by a key photosynthetic enzyme, Rubisco, that oscillates from CO_2_ assimilation to photorespiration and back [34,35]. The effects of monochromatic light and other unique artificial light treatments on the carbon isotope fractioning have not been investigated until now.

## 2. Materials and Methods

### 2.1. Plant Material

Lettuce, *Lactuca sativa* L., plants of the Aficion RZ cultivar were used in our studies. This is Batavia-type lettuce, leaves with strongly wavy edge, light green. Batavia lettuce is highly appreciated in the market due to the variability in shape, color, texture, and taste. As for the nutritional value, it is a source of vitamin A, niacin, riboflavin, thiamine, Ca, Fe, K, Mn, Se, and β-carotene [36]. Aficion cultivar is widely grown in greenhouses and vertical farms with artificial lighting.

### 2.2. Cultivation Conditions

**Figure 1:**
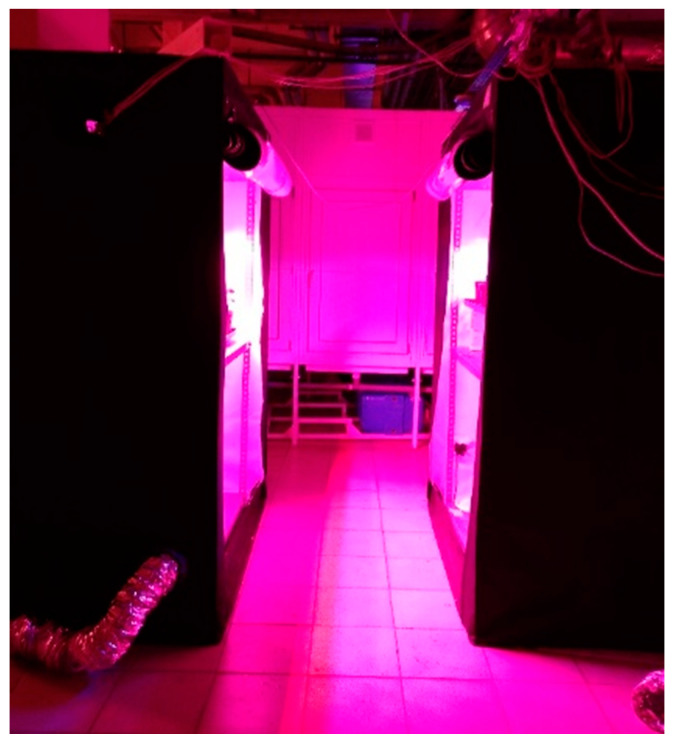
Plant-growing chambers with various light environments.

Plants were grown in growth chambers (Urbangrower 150, China; Figure 1) with various light treatments according to experimental layout described in Section 2.3. Each chamber had dimensions of 1.50 × 0.90 × 2.00, 2.7 m^3^, with gloss white walls. Chambers were supplied with fans; day/night temperature was 20/18 °C, with less than 1 °C variation over time and 1 °C variation among chambers.

Plants were grown in 2 L vegetational vessels (3 plants in each container). Seeds were sown directly into the commercial neutralized peat-based substrate “Agrobalt-C” (Pindstrup, Pskov region, Russia) with pH 6.0–6.5 and complete macro- and micronutrient supply including 150 mg L^−1^ [NH_4_^+^ andNO_3_^−^], 270 mg L^−1^ P_2_O_5_, and 300 mg L^−1^ K_2_O. Substrate humidity was maintained at 70% of full water capacity, watering up to calculated weight.

### 2.3. Light Treatments

**Figure 2:**
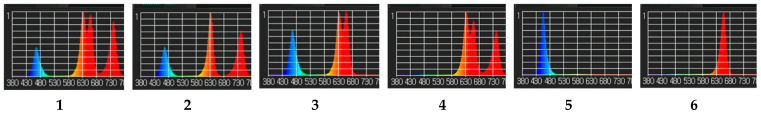
Light treatments: (**1**) “460 + 640 + 660 + 730”—4-peak reference treatment; (**2**) “460 + 640 + 730”—3-peak treatment missing red-light R_660_ region; (**3**) “460 + 640 + 660”—3-peak treatment missing far-red-light FR_730_ region; (**4**) “640 + 660 + 730”—3-peak treatment missing blue-light B_460_ region; (**5**) “450”—monochromatic blue-light B_450_ region; (**6**) “659” monochromatic red-light R_659_ region.

Plant chambers were illuminated with lamps consisting of various light-emitting diode (LED) bars specifically designed to provide a custom spectrum in each chamber. Fixtures consisted of light modules with tunable light-emitting diodes varying in wavelength and spectral composition of the emitted light over wide ranges (Figure 2).

Four types of high-performance narrow-band 3-Watt LEDs (Estar Technology, Changchun, China) were used: short-wave red (∆λ_0.5_ = 623 ÷ 641 nm, λ_max_ = 632 nm), long-wave red (∆λ_0.5_ = 646 ÷ 674 nm, λ_max_ = 660 nm), far-red (∆λ_0.5_ = 727 ÷ 751 nm, λ_max_ = 739 nm), and blue (∆λ_0.5_ = 452 ÷ 477 nm, λ_max_ = 465 nm). The control light treatment included all 4 types of LEDs, and in each of the other regimes one of them was excluded (except short-wave red) in order to elucidate the wavelength that affected distinct crop physiological processes. Short-wave red was used as an additional background spectral region to provide chlorophyll *a* excitation in the absence of long-wave red light. The same daily light integral (DLI) of 9.72 mol m^−2^ d^−1^ was maintained in all the treatments with photosynthetic photon flux density (PPFD) 150 µmol m^−2^ s^−1^, photoperiod 18 h. Spectra of the resulting lamp systems were measured with a spectrometer UPRtek PG100N (Taiwan). To measure the PPFD in the PAR region, an LI-191R quantum sensor with an LI-250A data logger (LI-COR Biosciences, NE, USA) was used. It was measured at the top of the plant canopy (the distance from the light source was ≥50 cm), and each chamber was adjusted to maintain PPFD at ±5%. To provide uniform PPFD, plant pots were moved and rotated within the marked uniform light platform every second day.

In the experiment on the red and blue monochromatic light effects, two types of tunable LEDs (Cree, USA) were used: red (∆λ_0.5_ = 647 ÷ 671 nm, λ_max_ = 659 nm) and blue (∆λ_0.5_ = 438 ÷ 462 nm, λ_max_ = 450 nm).

To provide easy reading of the figure legends, wavelengths representing figures for combined-spectra regions (blue, short-wave red, long-wave red, and far-red) are “rounded” to 460, 640, 660, and 730, respectively.

### 2.4. Plant Growth Parameters Analyses

Four plants per each treatment were destructively harvested 30 days after emergence. The number of leaves (>1 cm) per plant was counted, and total leaf area was measured using a leaf area meter LI-3000A (LI-COR Biosciences, NE, USA). Shoot fresh weight was measured using an electronic balance. Subsequently, shoots were oven-dried to a constant weight at 70 °C for dry weight determination. Specific leaf weight (SLW) was calculated by dividing leaf weight by leaf area (dry weight per unit leaf area).

### 2.5. Photosynthesis and Transpiration

Plant leaf photosynthetic rate and transpiration analyses were carried out using an LI-6400XT Portable Photosynthesis System (LI-COR Biosciences, NE, USA) with a standard leaf chamber of 2 × 3 cm. During the measurements, CO_2_ concentration was maintained at 400 ± 12.0 µmol mol^−1^, air temperature 21–23 °C, and air humidity 60 ± 4.0%.

Photosynthesis and transpiration were measured at the same light intensity as the growth light. Photosynthetic water use efficiency was calculated as the ratio of the rate of carbon assimilation (photosynthesis) to the rate of transpiration.

The light response curve, i.e., the photosynthetic rate as a function of incident light intensity, was measured for four different leaves using an infrared gas analyzer (IRGA) with an LI-6400XT standard lighting chamber; the internal LED lamp in the IRGA machine was used as a light source.

### 2.6. Chlorophyll a Fluorescence Determination

Chlorophyll *a* fluorescence in PSII was measured using Junior-PAM fluorimeter (Heinz Walz, Germany). Minimum (Fo) and maximum (Fm) fluorescence rates were determined after 15 min leaf exposition in darkness. Maximum quantum efficiency of PSII Fv − Fm = (Fm − Fo)/Fm was calculated after [37]. Relative PSII operating efficiency (ΦPSII) of the light-adapted leaves was calculated as ΦPSII = (F′m − Ft)/F′m. Chlorophyll a non-photosynthetic quenching (NPQ) was calculated as NPQ = (Fm − F′m)/F′m. Photochemical electron transport rate (ETR) was calculated as ETR = (Φ_PSII_)·PPFD·0.5. Fluorescence parameters were determined in 4–6 biological replicates.

### 2.7. Carbon Isotope Discrimination (Δ) Measurements

Plant sampling was conducted with 6 h intervals during 24 h cycle: at the start of 18 h photoperiod and at 6, 12, and 18 h. Plant leaves were sampled in 4 replicates and dried in an oven at 65 °C.

Dry plant material was milled using Vibromill vibrator. After milling in powder, samples were weighed in tin containers and introduced by means of an autosampler into the elemental analyzer (FlashEA, Thermo Electron, Waltham, MA, USA), where in presence of O_2_ and catalysts, they were quantitatively burnt to CO_2_. The formed gas was separated from other combustion gases on a chromatographic column and transferred via ConFlo interface to the isotope ratio mass-spectrometer (Delta V, Thermo) for analysis. Each sample was analyzed three times.

For calibration of IRMS instrument and control of results’ accuracy we used IAEA standards of L-glutamic acid (USGS40, USGS41).

The values of the isotope ratio are expressed in δ‰ according to the formula

$$\delta ‰=\left(R_{\mathrm{sample}}-R_{\mathrm{standard}}/R_{\mathrm{standard}}\right)\times 1000$$

where R_sample_ and R_standard_ are the ^13^C/^12^C ratios of the sample and the standard, respectively. By international convention, the standard used for the analysis was the carbon belemnite from the PeeDee formation (VPDB).

### 2.8. Statistical Analysis of Experimental Data

For each light treatment, four replications were tested during plant phenotyping (sampling). Statistical analysis of physiological parameters was performed using one-way analysis of variance (ANOVA) followed by Duncans’ multiple range test with MS Excel software and AGROS software (version 2.11, Moscow, Russia). In the graphs, means ± standard errors (SE) are presented; means followed by the same letter were not different at *p* ≤ 0.05.

## 3. Results

### 3.1. Growth Responses

**Figure 3:**
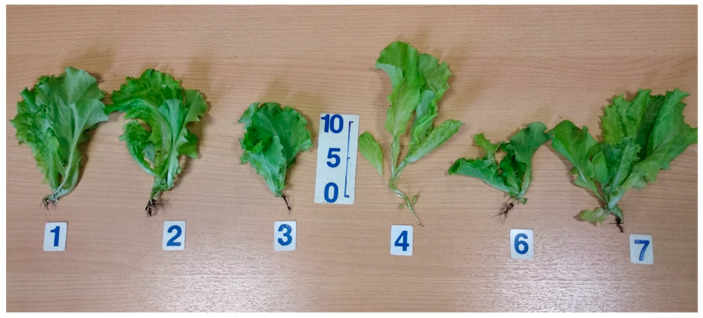
Representative photo of plants from each light treatment, 20 days after emergence: (1) “460 + 640 + 660 + 730”—4-peak reference treatment; (2) “460 + 640 + 730”—3-peak treatment missing red-light R_660_ region; (3) “460 + 640 + 660”—3-peak treatment missing far-red-light FR_730_ region; (4) “640 + 660 + 730”—3-peak treatment missing blue-light B_460_ region; (6) “450”—monochromatic blue-light B_450_ region; (7) “659” monochromatic red-light R_659_ region.

**Figure 4:**
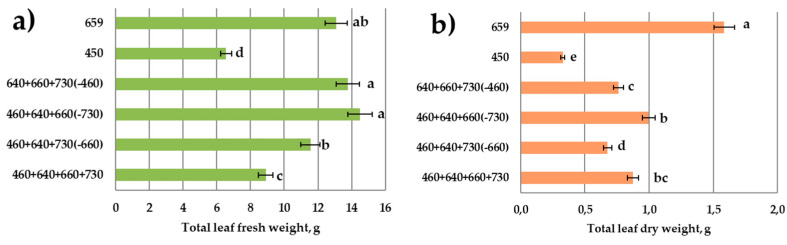

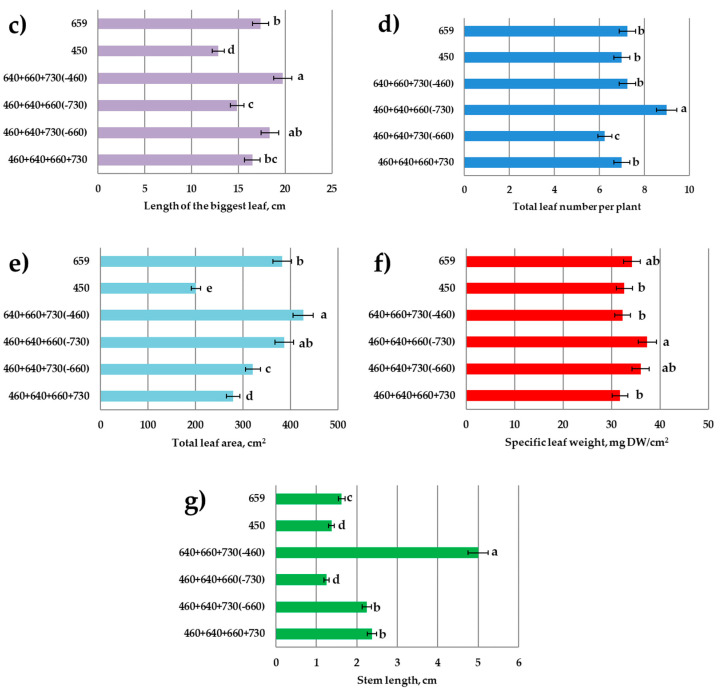
Growth parameters of lettuce plants in response to various light treatments. Sampling 30 days after emergence. Means ± standard error (SE); means followed by the same letter were not different at *p* ≤ 0.05. (**a**) Total leaf fresh weight; (**b**) total leaf dry weight; (**c**) length of the biggest leaf; (**d**) total leaf number per plant; (**e**) total leaf area; (**f**) specific leaf weight; (**g**) stem length. Light treatments from the bottom of *y*-axis: “460 + 640 + 660 + 730”—4-peak reference treatment; “460 + 640 + 730”—3-peak treatment missing red-light R_660_ region; “460 + 640 + 660”—3-peak treatment missing far-red-light FR_730_ region; “640 + 660 + 730”—3-peak treatment missing blue-light B_460_ region; “450”—monochromatic blue-light B_450_ region; “659” monochromatic red-light R_659_ region.

Lettuce plant growth (leaf biomass accumulation) was significantly retarded in the monochromatic blue light (Figure 3 and Figure 4). It was also reduced in the combined-spectrum environment missing blue light. Monochromatic red light was especially favorable for biomass accumulation. Red light’s absence was unfavorable for dry biomass accumulation, though it did not affect fresh biomass yield.

In the treatment missing blue light, plants demonstrated a tendency towards bolting. The highest bolting resistance was observed in response to single-blue-light treatment or to the combined spectrum missing far-red light. Additionally, leaf size was reduced in the single-blue-light treatment and in response to the combined spectrum missing far-red light. In the blue-light treatment, leaf size reduction resulted in dramatically reduced total leaf area. However, in the combined spectrum missing far-red light, there was no reduction in leaf area due to the increased total leaf number; also, the highest specific leaf weight was observed under these conditions. Blue-light knockout in the combined spectrum resulted in a considerable leaf area increase.

### 3.2. Photosynthesis and Transpiration

**Figure 5:**
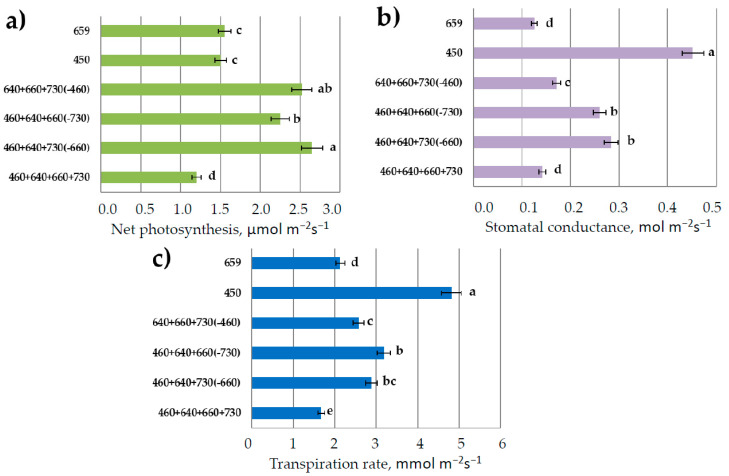
CO_2_—H_2_O leaf exchange in lettuce plants in response to various light treatments. (**a**) Net photosynthesis; (**b**) stomatal conductance; (**c**) transpiration rate. Means ± standard error (SE); means followed by the same letter were not different at *p* ≤ 0.05. For light treatments legend see Figure 4.

The highest net photosynthesis was observed in plants grown in the combined-spectrum light environment missing red or blue light (Figure 5). Interestingly, the net photosynthesis in the reference treatment was lower than in all the other treatments.

It has been shown in previous studies that blue and red light induce stomatal opening via different pathways [38]. In our experiment, the highest stomatal conductance and transpiration were observed under monochromatic blue light. Red- or far-red-light absence in the combined spectrum decreased these parameters as compared to blue-light treatment, though more significant response was observed in the absence of blue light. Water use efficiency (WUE, photosynthesis/transpiration ratio) was extremely low under blue light (mostly due to the highest transpiration rate) and increased by three times in the treatments with red or blue light. 

**Figure 6:**
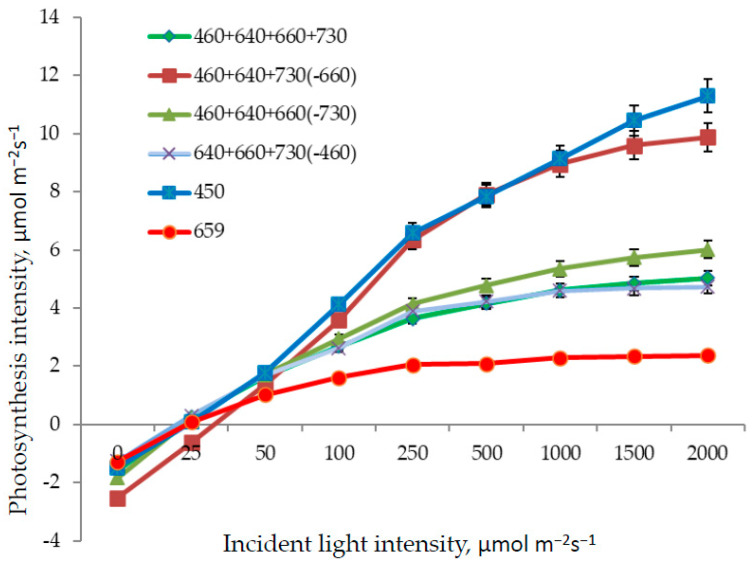
Light response curves in lettuce plants in response to various light treatments Means ± standard error (SE). For light treatments legend see Figure 4.

As for the light response curve determination, the lowest photosynthesis intensity at saturating PPFD was observed in response to red light (Figure 6). Here, low light intensity at light response curve saturation was found, as well. This kind of response is typical for plants originating from the shaded habitats. The highest photosynthesis at saturating light intensity was observed in response to blue light and the combined spectrum without red light R_660_; in part, the absence of the long-wave red light was compensated for here by short-wave red light R_640_.

### 3.3. Chlorophyll a Fluorescence

**Figure 7:**
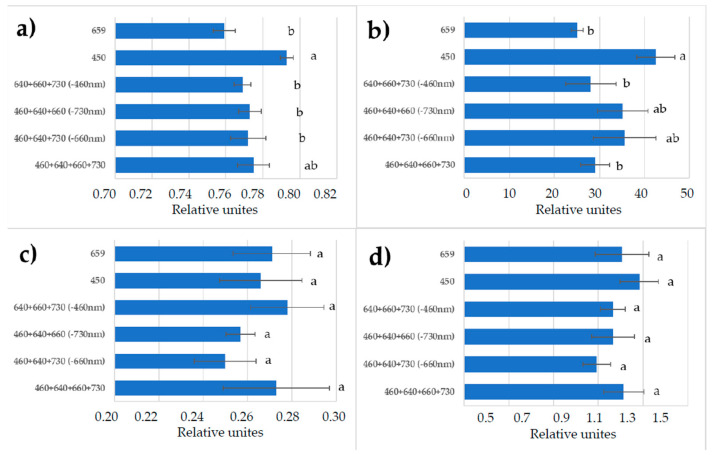
(**a**) Maximum quantum efficiency (Fv/Fm) of photosystem II (PSII); (**b**) photochemical electron transport rate (ETR); (**c**) relative PSII operating efficiency (ΦPSII); (**d**) chlorophyll *a* non-photosynthetic quenching (NPQ) in the leaves of lettuce plants in response to various light treatments. Means ± standard error (SE); means followed by the same letter were not different at *p* ≤ 0.05. For light treatments legend see Figure 4.

The maximum quantum efficiency of PSII photochemistry (Fv/Fm) was comparable in all the red + blue spectral treatments and single red (Figure 7). Monochromatic blue light favored the increase in Fv/Fm. There were variations in the level of relative operating efficiency of PSII, but the differences among the treatments were not significant. Higher effectiveness of the photochemical processes was observed in response to monochromatic blue light and in treatments without red or far-red light (changes of the photochemical electron transport, ETR). Chlorophyll *a* non-photosynthetic quenching (NPQ) was relatively higher in the monochromatic-blue-light treatment.

### 3.4. Carbon Isotopes Discrimination

**Figure 8:**
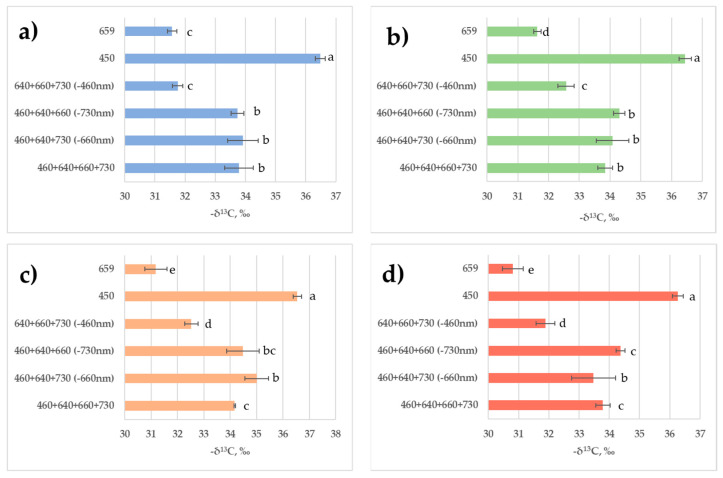
Carbon isotope composition of the leaves in lettuce plants grown in various light environments during 24 h cycle. Carbon isotope composition is given in PDBV δ^13^C units. Means ± standard error (SE); means followed by the same letter were not different at *p* ≤ 0.05. (**a**) After 6 h of illumination; (**b**) after 12 h of illumination; (**c**) after 18 h of illumination; (**d**) at the end of night after 6 h of darkness. For light treatments legend see Figure 4.

Sampling of plant material was carried out with 6 h intervals. Different light treatments showed multidirectional effects on the carbon isotope composition of leaf biomass, resulting in isotopic shifts in opposite directions (Figure 8). The strongest effects were observed in plants in response to monochromatic red and blue light as compared to the combined reference spectrum. Isotopic changes occurred in opposite directions. Thus, blue-light treatment resulted in ^12^C enrichment of the leaf biomass; after 6 h of illumination it was 2.56‰ “lighter” in relation to the biomass in control treatment. Red light, on the contrary, induced ^13^C enrichment of the leaf biomass; after 6 h of illumination it was 2.34‰ “heavier” in relation to the biomass in control experiment. In all the other treatments with combined spectrum, blue lightpresence in the spectrum resulted in a stable isotopic shift towards the enrichment of biomass with the ^12^C isotope. Additionally, in a combined-spectrum environment missing blue light, the presence of red light resulted in biomass enrichment with the ^13^C isotope.

The results of the carbon isotopic differences in leaf biomass during the transition from light to dark are of particular interest. One could expect a strong rearrangement of the metabolic fluxes between day and night periods. Indeed, as we can see from Figure 8, during the light period, the leaf biomass became enriched with the ^12^C isotope as compared to the biomass carbon composition detected at the end of the dark period. These isotopic differences occurred in all the lighting modes. Isotopic differences were quite distinct though not very great.

The data obtained are consistent with the results of Gessler et al. [39], who studied daily variations in the carbon isotope composition of the *Ricinus communis* plants and had found similar daily variations not only in the carbon of the plant leaf biomass but also in the carbon of its water-soluble and water-insoluble fractions and phloem sap.

## 4. Discussion

Studies on the light action in plants using various applications of LED techniques provide new insights into plant photobiology. The plant photosynthesis action spectrum matches with blue and red regions of photosynthetically active radiation in a natural environment [40,41,42]. In an artificial-light environment, the joint application of red and blue light usually results in increased plant photosynthesis and productivity [11,12,22]. Additionally, blue light is thought to participate in the acclimation of leaf photosynthesis to irradiance during growth [10,43]. These two spectral regions were the basic variables in our photobiological studies.

In our experimental set-up, we applied combined-spectrum treatments within two ranges of red light (R_640_, R_660_) trying to separate direct light effects on the PSA and light-induced photomorphogenetic responses controlled by the phytochromes. Indeed, far-red-light absence in the combined spectrum resulted in axial organ growth inhibition as compared to the treatments with far-red light (Figure 4g). Leaf blade elongation was also retarded (Figure 4c) due to the blocking of phytochrome-mediated shade-avoidance syndrome. Interestingly, the total leaf number increased significantly in this treatment, providing the growth of the light-harvesting leaf area of the plant. It is still unclear whether this response was observed due to the decreased plastochrone in the FR-deficient treatment or if other more sophisticated compensation mechanisms were involved. Similar results with stem and leaf growth inhibition were observed in response to monochromatic blue light (Figure 4c,g). Actually, the most serious inhibition of leaf blade growth in comparison with the other treatments was found in response to blue light. A reduction in leaf growth in response to blue light decreased plant biomass accumulation significantly.

Data on the decreased total leaf fresh weight yield in the treatment combining all four spectral regions in comparison with monochromatic red were unexpected. However, there are other data suggesting that lettuce biomass under monochromatic red was greater than under mixed red and blue light [44]. Comparable responses in other species were observed by Wollaeger and Runkle [45]. So, the synergetic or antagonistic effects of red and blue light on lettuce are still confused, and more studies need to be conducted [46].

As far as plant growth was inhibited in monochromatic blue light, net photosynthesis was also at a low rate in comparison with the combined-spectra treatments missing distinct spectral regions. The photosynthesis rate in the treatment missing long-wave red light R_660_ was one of the highest due to the compensation by short-wave red R_640_; photosynthesis at saturating light intensity (light response curve) was also very high (Figure 5).

Net photosynthesis in the reference treatment was lower than in all the other treatments. This is most likely because in more stressful environments lacking distinct spectral regions, compensation mechanisms were activated. Additionally, the red-light PPFD share in the combined-spectrum treatment was much lower than PPFD in the monochromatic-red-light treatment. On the other hand, we observed plant acclimation to the abnormal light environments as a long-term process (sampling 30 days after emergence). This is most likely because an increased assimilate demand and increased sink capacity were the drivers of photosynthesis in monochromatic red light. We shall try to investigate this phenomenon in the future studies. Blue-light treatment significantly increased stomatal conductance and transpiration rate in plants and decreased their WUE; comparable results were obtained in tomato plants [47].

The main points of carbon isotope fractionation during photosynthesis are located at the crossings of the central metabolic pathways; therefore, the isotopic effects are reflected in the carbon isotope composition of biomass, fractions, and of the overwhelming number of metabolites [35]. The first carbon isotope fractionation point is located at the entry of the pentose phosphate reduction cycle (Calvin cycle) and is associated with the reaction of enzymatic carboxylation of ribulosebisphosphate (RuBP). As a result, the assimilated carbon atoms are enriched in ^12^C in relation to the environmental CO_2_. The enzyme that controls carboxylation, Rubisco, has the properties of oxygenase and is able to simultaneously redirect a part of the carbon flux assimilated in Calvin cycle to glycolate cycle, where it is partly oxidized to CO_2_ and released back into the environment, creating so-called photorespiration flux. A probable mechanism of switching the functions of the enzyme is maintained by the changing ratio of CO_2_/O_2_ concentrations in the cell [48]. Due to such organization of photosynthesis, the activities of the Calvin cycle and glycolate cycle are separated in time, and the fluxes of carbon substrates resulting from assimilation and photorespiration become independent and discrete, that is, represented as separate portions [49]. In our experiment, we observed increased stomatal conductance in response to blue-light treatment (Figure 5). As a result, an increased CO_2_ supply could enhance Rubisco carboxylating activity and it was followed with leaf tissue ^12^C enrichment (Figure 8). These results are consistent with the data of other authors that have shown that δ^13^C correlates negatively with stomatal conductance [50].

The most intensive lettuce leaf tissue enrichment with ^13^C was observed in the treatments with monochromatic red light followed by in the combined-spectra environment missing blue light; in the last case this response could be attributed to the contradictory information from the blue- and red-light photoreceptors, as it was mentioned in Section 1. On the contrary, the biomass of plants subjected to blue-light treatment was enriched with the ^12^C isotope. We have to stress here that plants were subjected to the long-term (during the whole growing cycle) light treatment. Therefore, chloroplast genesis could be affected significantly in the absence of blue light, as it was observed earlier [51]. On the contrary, monochromatic blue light was more favorable for chloroplast development and functioning [20,51]. In our studies, monochromatic-blue-light treatment maintained better plant photosynthetic performance, i.e., the highest maximum quantum efficiency (Fv/Fm) and a higher electron transport rate (ETR). Data from the light response curves show that photosynthesis at saturating light intensity in the blue-light-grown plants was four times higher than in the red-light-grown plants. Taking into consideration the facts discussed above, a possible explanation could be based on the variability in plant adaptations to the abnormal light environments during long-term 30-day exposure. That has resulted in the disturbance of PSA but to a lesser extent in the case of blue-light treatment as compared to red-light treatment.

The second point of carbon isotope fractionation is connected with increased photorespiration when observations show that plant biomass becomes enriched with ^13^C [52]. This means that photorespiration is accompanied by an isotope effect of opposite sign than photoassimilation. Numerous studies on isotope fractionation in plants and artificial mutants have proved that the glycine decarboxylase reaction of the glycolate cycle was another place where the isotope effect is observed [33,53].

The third point of carbon isotope fractionation relates to post-photosynthetic metabolism and is associated with the end of the glycolytic chain where pyruvate dehydrogenase reaction proceeds. The observed proximity of the carbon isotope composition of the total plant biomass to assimilatory carbon pool suggests that the glycolytic chain and the majority of metabolites (lipids, proteins, lignins, and some carbohydrates), whose synthesis occurs via glycolytic chain, are supplied with the substrates of the assimilatory pool [54]. At the same time, the syntheses of soluble carbohydrates, organic acids, some amino acids, and other metabolite sis mainly bound to the “heavy” photorespiratory carbon pool. Because of the strict temporal and spatial organization in a cell, noticeable mixing of carbon fluxes does not occur, and various isotope distinctions exist [55].

The idea of the Rubisco oscillating mode of action has been analyzed extensively [32,34,35,56] and theoretically it was shown that oscillations can exist under real photosynthetic cell conditions. In the present paper, we returned to this idea. We assumed the presence of an isotopically “light” assimilatory pool and isotopically “heavy” pool of metabolites appear during photosynthesis as a result of dual function of Rubisco.

In our experiment, in all cases, leaf biomass at the end of light period was enriched in ^12^C as compared to the leaf biomass at the end of dark period. Isotopic differences were quite distinct though not very great. Possible explanations of these differences could be given from our earlier paper [57] based on the model of oscillatory photosynthesis discussed above. Plant tissues enrichment with ^12^C isotope during the light period was due to the fact that at this time lipids, proteins, lignins, and other structural components were synthesized mainly in the leaf. The isotopically “light” assimilatory pool was the substrate source for them. During the dark period, the outflow of assimilates to generative organs and heterotrophic tissues occurred. The outflow of assimilates occurred mainly in the form of sucrose and other water-soluble carbohydrates and metabolites, the isotope-heavy photorespiratory fund being their carbon source. Different sources of substrates for the synthesis of structural units and transport agents induced isotopic differences in daily variations of leaf biomass. Similar isotopic shifts were observed by other researchers while studying the isotopic differences between photosynthetic and heterotrophic organs and tissues [58].

We can conclude that blue light enhanced the assimilation function of the leaf, while red light enhanced the photorespiratory function. The simultaneous presence of blue and red light compensated for their mutual effects, and therefore the effects of light from the other spectral regions on the isotopic shifts became indistinguishable from the control. It was shown that duration of illumination (6, 12, and 18 h) had a weak effect on the isotope composition of biomass.

## 5. Conclusions

In our studies, variations in incident light spectral quality simulated with LEDs influenced growth and development in lettuce plants in several ways via direct effects on photosynthesis and control of plant photomorphogenetic responses. PSA structure and growth activity were significantly affected in the distinct light treatments, and these changes influenced source–sink relations in plants through the assimilate demand, etc. (indirect light effects on photosynthesis).

Our studies have shown that monochromatic blue light retarded lettuce plant growth, and monochromatic red light accelerated it. In plants exposed to blue light, the assimilating leaf area growth was retarded (both source and sink simultaneously!), and even an increased photosynthesis rate could not compensate for this delay. In the combined spectrum, far-red-light action was also important as far as it had triggered the shade-avoidance response and enhanced plant assimilate demand.

For the first time, it was found that the light of different PAR spectral regions affected the carbon isotope composition of leaf biomass. The strongest and most opposite in direction effects of monochromatic blue and red light were observed. Continuous blue-light treatment resulted in the ^12^C enrichment of lettuce plant leaf biomass by about 3‰, whereas continuous red-light treatment resulted in ^13^C enrichment of the same value. The effects of light of the other PAR spectral regions studied were considerably less significant. Daily variations in the leaf tissue carbon isotope composition were not significant.

Further research is needed to assess light-induced isotopic effects in plants and the mechanisms underlying them. These studies also could provide significant starting points for the development of the dynamic (changing in time) lighting regimes combining the advantages of the distinct spectra studied above at certain periods of plant growth. Thus, plant acclimation and photosynthetic improvements in response to added far-red and green-light wavelengths to the main red–blue spectrum have already been studied along with the changing red-to-blue-light ratio [59].

It is known that photorespiration can serve as an energy sink preventing the overreduction in the photosynthetic electron transport chain and photoinhibition, especially under stress conditions that lead to reduced rates of photosynthetic CO_2_ assimilation and provides metabolites for other metabolic processes, e.g., glycine for the synthesis of glutathione, which is also involved in stress protection [60,61,62]. Therefore, another area of interest could be studies on plant stress responses and stress tolerance mechanisms including light-induced stress, e.g., extremely high PPFD or abnormal spectral environment adaptation.

## Data Availability

Data sharing is not applicable to this article.
